# Treatment-Associated Neuroplastic Changes in People with Stroke-Associated Ataxia—An fMRI Study

**DOI:** 10.3390/neurolint17060084

**Published:** 2025-05-29

**Authors:** Patricia Meier, Christian Siedentopf, Lukas Mayer-Suess, Michael Knoflach, Stefan Kiechl, Gudrun Sylvest Schönherr, Astrid E. Grams, Elke R. Gizewski, Claudia Lamina, Malik Galijasevic, Ruth Steiger

**Affiliations:** 1 Department of Neurology, Medical University of Innsbruck, 6020 Innsbruck, Austria; lukas.mayer@i-med.ac.at (L.M.-S.); michael.knoflach@i-med.ac.at (M.K.); stefan.kiechl@i-med.ac.at (S.K.); 2 VASCage, Centre on Clinical Stroke Research, 6020 Innsbruck, Austria; 3 Clinical Department of Radiology, Medical University of Innsbruck, 6020 Innsbruck, Austria; christian.siedentopf@i-med.ac.at (C.S.); astrid.grams@i-med.ac.at (A.E.G.); elke.gizewski@i-med.ac.at (E.R.G.); malik.galijasevic@i-med.ac.at (M.G.); ruth.steiger@i-med.ac.at (R.S.); 4 Department of Neurology, University Hospital Innsbruck, 6020 Innsbruck, Austria; gudrun.schoenherr@tirol-kliniken.at; 5 Neuro Imaging Research Core Facility, Medical University of Innsbruck, 6020 Innsbruck, Austria; 6 Institute of Genetic Epidemiology, Medical University of Innsbruck, 6020 Innsbruck, Austria; claudia.lamina@i-med.ac.at

**Keywords:** acute stroke, ataxia, neurorehabilitation, coordination exercises, balance, plasticity, fMRI, foot tapping, motor imagery

## Abstract

**Background/Objectives**: In consideration of the significance of the pursuit of training-induced neuroplastic changes in the stroke population, who are reliant on neurorehabilitation treatment for the restoration of neuronal function, the objectives of this trial were to investigate fMRI paradigms for acute stroke patients with ataxic symptoms, to follow up on changes in motor function and balance due to recovery and rehabilitation, and to investigate the different effects of two treatment methods on neuronal plasticity. **Methods**: Therefore, fMRI-paradigms foot tapping and the motor imagery (MI) of a balancing task (tandem walking) were employed. **Results**: The paradigms investigated were suitable for ataxic stroke patients to monitor changes in neuroplasticity while revealing increased activity in the primary motor cortex (M1) and the cerebellum over 3 months of treatment. Furthermore, analysis of the more complex balance task revealed augmented activation of association areas due to training. Coordination exercises, constituting a specific treatment of ataxic symptoms, indicate more consolidated brain activations, corresponding to a faster motor learning process. Activation within Brodmann Area 7 has been prominent among all paradigms, indicating a special importance of this region for coordinative functions. **Conclusions**: Further studies are needed to confirm our results in larger patient groups. **Clinical Trial Registration**: German Clinical Trials Registry (drks.de). Identifier: DRKS00020825. Registered 16.07.2020.

## 1. Introduction

Stroke affects 16.9 million people annually, with ataxia being a common symptom when the vertebrobasilar system is impacted [1]. Infarctions of the superior cerebellar artery (SCA), posterior inferior cerebellar artery (PICA), or anterior inferior cerebellar artery (AICA) frequently lead to coordination deficits [2]. Ataxia can manifest in a variety of ways, including, but not limited to, balance loss, the impairment of gait (gait ataxia), dysarthria, and nystagmus. Severe balance impairment has been demonstrated to hinder daily activities and increase the risk of falls and fractures, thereby impacting the quality of life [3].

Individually adapted neurorehabilitation approaches are intended to target this issue by improving mobility, speeding up reintegration into work, and enhancing the quality of life. However, the existing body of research on physical therapy (PT) interventions for post-stroke ataxia remains limited [4,5,6]. Previous studies have explored a variety of training methods, including treadmill training [7], robot-assisted gait training [8,9], and trunk exercises [10]. However, the incorporation of imaging techniques to investigate neuroplastic changes remains a rarity [7].

The employment of modern imaging techniques is of substantial importance in the context of rehabilitation trials as they facilitate the monitoring of neuroplastic changes, which do not solely occur initiated by damage (injury-induced neuronal plasticity) but also occur as a consequence of training. Training-induced neuronal plasticity describes any chemical or structural changes in the nervous system that are promoted by an intensified use of the affected extremities within the framework of a motor learning process [11], leading to an increase in motor performance [12].

Neuroplastic changes such as a posterior shift [13], a reduction in the hyperactivity of the association areas [14], or the lateralization to the lesioned hemisphere [15] can be observed, depending on the lesion size. Long-term potentiation (LTP) has been identified as the basic mechanism underlying the process of restructuring [16], which occurs during the distinct stages of motor learning (i.e., cognitive, associative, and autonomous stage) [14]. The role of the cerebellum in the motor learning process is crucial. The function of the cerebellum is to facilitate the acquisition and refinement of movements during the cognitive and associative phases of learning. In addition, the cerebellum plays a pivotal role in the automatization and retrieval of movement during the autonomous stage of learning [17]. However, it is important to note that cerebellar activity is reduced in the autonomous stage of learning in comparison with earlier phases [18]. Therefore, patients with cerebellar damage due to stroke experience limitations in motor learning, particularly in the context of trial-and-error learning. As compensation must be sought for patients with cerebellar damage, i.e., by the increased repetition of movements and intensive training [19] or gradual exposure to errors [20], coordination exercises have been identified as a method fulfilling these principles. Indeed, such exercises have been shown to facilitate recovery in patients diagnosed with degenerative cerebellar disease [21], a phenomenon that may be attributable to the unique nature of their approach. Yet, to the best of our knowledge, no imaging trial had ever been conducted for this specific treatment method to follow up on neuronal plasticity to monitor its effectiveness regarding motor learning.

To address this issue and the lack of knowledge of efficient PT treatment methods for patients with acute stroke and ataxic symptoms, a large randomized controlled trial (RCT) on the treatment of balance and walking was conducted [22] after the safety and feasibility for this population was confirmed [23]. To investigate the effects of treatment, especially of coordination exercises, on brain plasticity, neuronal recovery was monitored in a subgroup of patients by fMRI examination.

The hypothesis was formed that movement-relevant brain areas (i.e., the primary motor cortex, premotor and supplementary motor areas, cerebellum, and prefrontal cortex) are subject to differential activation depending on the therapy method.

Therefore, the aims of the inclusion of an fMRI examination into the RCT with acute stroke patients with ataxia were as follows:

1. To verify existing fMRI motor paradigms for their usability in this specific population: Considering the relatively uninvestigated population, the first aim of this trial was to investigate the usefulness of fMRI paradigms, such as foot tapping and the motor imagery (MI) of a balancing task, for this specific population and outcome.
2. To follow up on neuroplastic changes occurring due to four weeks of intensive supervised practice and eight weeks of home-exercise training, given the importance of a better understanding of neuronal recovery in stroke patients in general.
3. To investigate the effects of different approaches in physiotherapy practice, especially of coordination exercises, as they are thought to compensate for difficulties in motor learning due to cerebellar damage according to their exercise structure.

## 2. Methods

### 2.1. Participants and Study Design

This MRI examination was part of a larger randomized controlled trial aiming to investigate the effects of coordination exercises on balance and walking, including 51 acute stroke patients with ataxic symptoms (GLAAS II trial, [22]). The trial was conducted between 20 July 2020 and 30 June 2024 at the Clinical Department of Neurology, Medical University of Innsbruck, Austria, while the MRI examination for this trial was performed in cooperation with the Clinical Department of Radiology and the Neuro Imaging Research Core Facility, Medical University of Innsbruck, Innsbruck, Austria.

The study was conducted in accordance with the Declaration of Helsinki and approved by the Ethics Committee of the Medical University of Innsbruck, Austria (reference number 1050/2020, 2 July 2020). The approval of the ethics committee for the Amendment (MRI examination) was obtained on 9 February 2023. Written informed consent was obtained from all subjects involved in the study. Clinical Trial Registration was completed prior to study’s start at German Clinical Trials Registry (drks.de; Identifier: DRKS00020825; registered 16 July 2020).

Eligibility criteria for the MRI sub-study, compared to eligibility criteria of the main trial (Table S1, [22]), added “the presence of a cerebellar infarction” for inclusion criteria and the “presence of contraindications for an MRI examination” to exclusion criteria.

After the approval of the amendment for the additional MRI examination, 11 participants agreed, and 9 patients (Table 1) could be included in the additional MRI examination (1 participant could not tolerate the MRI examination and 1 participant did not have a cerebellar infarction).

Individuals in the GLAAS II trial either received coordination exercises (IG, Intervention Group) or standard physiotherapy (CG, Control Group). A comprehensive description of the interventions is provided in the open-access published study protocol [22], accompanied by a TIDieR checklist and an exercise program for each group (supplementary data sheets). Data was collected at baseline (T0), after 4 weeks of supervised training (T1), and after a further 8 weeks of independent home training (T2) according to an individualized exercise plan [22].

**Figure 1:**
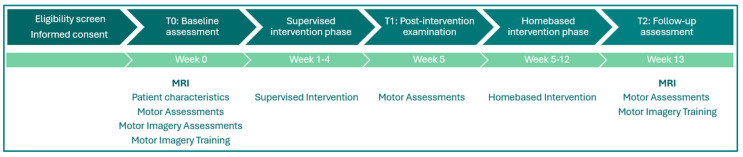
Study flow for one patient: MRI at T0 and T2.

Participants that agreed to participate in the MRI examination received this additional examination at baseline (T0) and after 3 months of training (T2) (Figure 1).

After each patient had been informed and consented to participate in the study, an MI ability test was carried out to test for the individuals’ MI capacity needed for the fMRI of the balancing task (Section 2.3) since it is known that the ability to visualize a movement can vary throughout patients. MI capability was assessed at the first appointment (T0), prior to the MRI examination [22], using two established assessments (KVIQ-10: Kinesthetic and Visual Imagery Questionnaire [24]; 6-Meter Walk Test: mental chronometry [25]), which have been validated for stroke patients [26,27,28,29,30,31]. Additionally, patients received a 20 min MI training session prior to each MRI examination.

### 2.2. Data Acquisition

All patients were examined in a 3T whole-body system (Skyra, Siemens Medical AG, Erlangen, Germany) incorporating a 64-channel head-neck coil (Siemens Medical AG, Erlangen, Germany). For diagnostical reasons structural imaging was performed first, including an isotropic transverse-oriented 3D T1-weighted MPRAGE (multiplanar reconstruction) with a voxel size of 0.8 × 0.8 × 0.8 mm^3^ (TR = 1690 ms, TE = 2.12 ms, FA = 8°, field of view FOV = 230 mm^3^, time of acquisition TA = 5:46 min), a transverse-oriented Turbo inversion recovery magnitude (TIRM) sequence (voxel size 0.7 × 0.7 × 3 mm^3^, TR = 8000 ms TE = 91 ms, FOV = 220 mm, TA = 3:14 min), a T2 weighted Turbo spin echo (TSE) (voxel size 0.6 × 0.6 × 2 mm^3^, TR = 5800 ms TE = 95 ms, FOV = 220 mm, TA = 3:13 min), a transverse-oriented diffusion-tensor-weighted imaging (DTI) sequence with b-factors = 0 (8 multiple reference images) and 1000 s/mm^2^ with 64 diffusion directions and a voxel size of 2 × 2 × 2 mm^3^ (TR = 9900 ms, TE = 92 ms, FOV = 250 mm^2^, number of slices = 74, TA = 12:24 min), a susceptibility weighted imaging (SWI) sequence (voxel size 0.9 × 0.9 × 1.8 mm^3^, TR = 27 ms TE = 20 ms, FOV = 220 mm, TA = 2:42 min), and a Time-of-Flight (TOF) MRA without contrast agent (voxel size 0.4 × 0.4 × 0.7 mm^3^, TR = 22 ms TE = 3.69 ms, FOV = 200 mm, TA = 3:58 min). Consequently, the functional echo-planar imaging EPI sequences (voxel size 2 × 2 × 3 mm^3^, TR = 2500 ms TE = 30 ms, FOV = 192 mm, number of slices = 38, TA = 4:29 min) were gained.

### 2.3. fMRI Design

**Figure 2:**
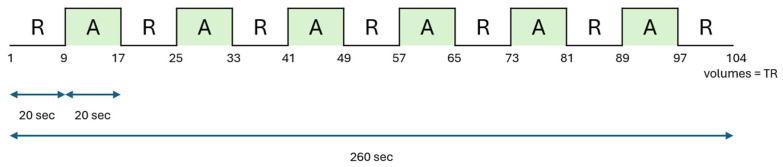
A block design is used for functional motor mapping: A = task activation; R = rest. A total of 104 functional images (volumes) were measured, with eight volumes per block.

We used a typical motor function MRI block design paradigm with 20 s of motor activity and 20 s of rest, where one run consisted of seven rest and six task blocks (Figure 2). Patients were given two different functional tasks during the examination: foot tapping, defined as a rhythmic up-and-down movement of the forefoot (ankle dorsiflexion), with left and right foot acquired separately, and subsequently, the MI of a balancing task.

MI (motor or mental imagery) of the balancing task, specified as tandem walking on a straight line, was chosen additionally to the more established tapping task because the primary outcome of the main trial was balance according to the Berg Balance Scale (BBS) [32]. MI is a special method that is frequently utilized in physiotherapy practice and was chosen as balancing cannot be performed actively in the scanner [33]. This choice also offered the advantage of expecting lower number of movement artifacts, compared to the foot tapping task, because it is only a visualization (MI) of movement.

**Figure 3:**
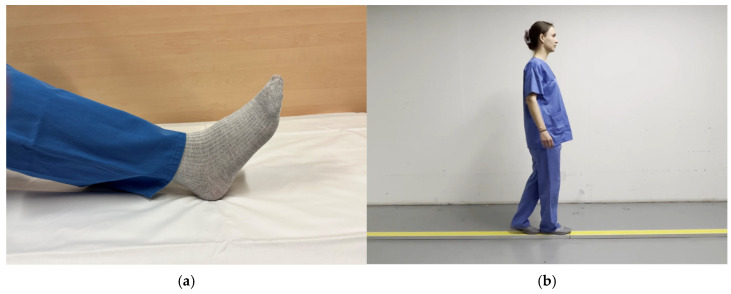
Functional magnetic resonance imaging paradigm—task activation: (**a**) image to prompt the foot tapping task; (**b**) video to prompt MI of the balancing task.

To enhance the activation of balance-relevant brain areas, action observation (watching a video of the balancing task) was chosen in addition to motor imagery (AOMI) [34,35]. Therefore, participants were shown a video of a person doing the balance task as a trigger to start the MI of the balancing task. A picture of a foot was displayed to prompt the foot tapping task. A standardized video and a standardized picture were shown to all patients (Figure 3) [22]. Resting phases were indicated by a “stop-sign” for foot tapping and a fixation cross for the MI of the balancing task.

The picture and video stimuli were displayed via video goggles (NordicNeuroLab, Bergen, Norway), mounted directly at the 64-channel head coil, and were presented in a block design with nordicAktiva software 1.2.1 (NordicNeuroLab, Bergen, Norway).

Patients were regularly informed about the upcoming paradigm. Furthermore, communication was important to maintain awareness of their understanding of the task, to provide feedback on their performance, and to ensure their physical well-being during the 45 min overall MRI scan time as these were acute stroke patients.

### 2.4. Data Analysis

All fMRI data were pre-processed using SPM 12 (Wellcome Department of Cognitive Neurology, London, UK) based on Matlab R2023b (The MathWorks Inc. Natick, MA, USA). Functional data were normalized using the transformation parameters of the anatomical image and smoothed with an 8 mm Full Width at Half Maximum (FWHM) Gaussian kernel. Statistical analysis of fMRI data uses a mass-univariate approach based on the general linear model (GLM) as implemented in SPM12. It compromises the following steps: (1) specification of the GLM design matrix, fMRI data files, and filtering; (2) estimation of GLM parameters using classical approach; and (3) interrogation of results using contrast vectors to produce Statistical Parametric Maps (SPMs). The task blocks were modelled with a box car function convolved with the canonical form of the hemodynamic response function. A high-pass filter (cut-off frequency: 128 Hz) was used to remove low frequency drifts. In an initial analysis aiming to identify blood-oxygenation-level-dependent (BOLD) responses in each group, an initial threshold of *p* < 0.001 on voxel level, family-wise error (FWE)-corrected, was used. In the analysis conducted to test for group differences between timepoints and treatment groups, a more liberal initial threshold of *p* < 0.05 on voxel level, FWE-corrected, was applied. Due to the small number of study participants, a first-level analysis was performed, with a simple one-dimensional T-contrast, producing summary data of our population.

## 3. Results

### 3.1. Utility of Chosen fMRI Paradigms in Acute Stroke Patients with Ataxic Symptoms

**Figure 4:**
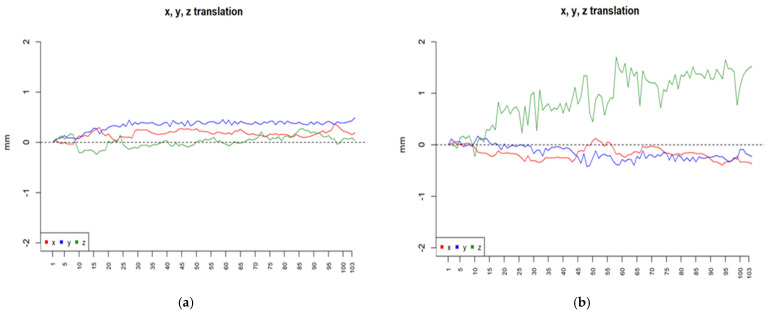
Example of translational (x, y, z) movement artifacts for foot tapping with the left foot: (**a**) from a patient with severe ataxia (SARA 20.5 points); (**b**) from a patient with moderate ataxia (SARA 9.5 points).

The MRI protocol and fMRI design utilized in this study both demonstrated good feasibility for patients suffering from acute strokes and ataxic symptoms. Patients demonstrated commendable compliance, attending all scheduled examinations and exhibiting excellent tolerance for the duration of the scanning procedure. They successfully completed all tasks without encountering any difficulties. However, a few minor issues occurred as patients initiated the foot tapping task on the contralateral side, deviating from the protocol-defined procedure. In this instance, the paradigm was halted and reinitiated to ensure that the labelling of the acquired MRI sequence (functional EPI) corresponded with the actual task that had been performed. The high level of compliance observed during the examination may have been attributable to the study’s design, wherein only patients with none or mild cognitive impairment were enrolled. Furthermore, it can be assumed that the provision of constant communication with the participants during the intermissions between paradigms might have been beneficial. To ensure the effective management of motion artefacts, the 64-channel head-neck coil of the scanner was equipped with supplementary head positioning pads with the objective of enhancing both comfort and stability. Nevertheless, when inspecting movement artifacts occurring during fMRI, two participants had to be excluded for the further analysis of the foot tapping tasks (participants 01 and 05 for the left side; participants 05 and 09 for the right side) at both measurement timepoints due to rhythmic activation and/or excessive head movement (more than 3 mm). When examining the movement artifacts in more detail, no relation between the severeness of ataxic symptoms and the intensity of the rotational or translational shifting of the head during the scan could be observed (Figure 4). It is considered to be a standard occurrence that movement artefacts emerge, thus rendering fMRIs unsuitable for analysis. The absence of correlation between ataxic symptoms and movement artefacts further substantiates the conclusion that acute stroke patients with even severe ataxic symptoms, characterized by pronounced and uncontrollable movements, are as suitable for fMRI examination as patients with supratentorial strokes.

The MI of the balance task revealed fewer artifacts due to movement; nevertheless, one patient had to be excluded for further analysis due to insufficient motor imagery skills (KVIQ-10), which was confirmed by the single-subject analysis, where only a few non-significant and not-conclusive brain regions were activated.

**Figure 5:**
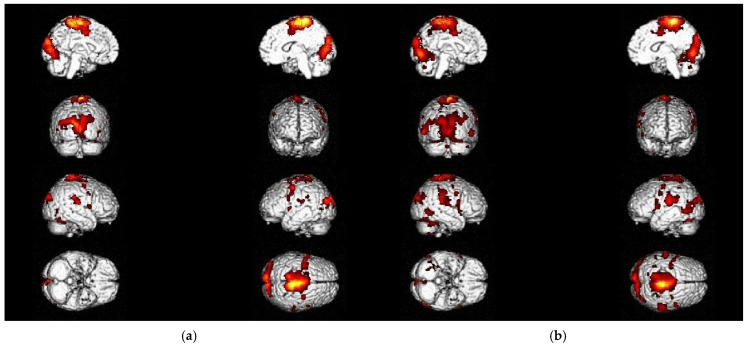
Activation patterns during foot tapping task (left side) in stroke patients with ataxia: (**a**) at T0; (**b**) at T2.

The main activations for the foot tapping task of both sides (P_corr_ < FWE 0.001) at T0 and T2 were observed in the somatosensory (S1) and primary motor cortex (M1) of the lower extremities, as well as in the premotor cortex (PMC), the supplementary motor area (SMA), and the supramarginal gyrus (SMG). Besides these movement-relevant brain regions, visual and language-relevant areas, like the secondary and tertiary visual cortex (V2, V3), the fusiform gyrus (FG), and the Broca Area, were active during this motor paradigm (Figure 5). Detailed information about Brodmann Areas (BAs), coordinates, and clusters above the threshold is listed in the Supplementary Materials for the foot tapping task at T0 for the left (Table S2) and right sides (Table S3), as well as at T2 for the left (Table S4) and right feet (Table S5).

These findings were consistent when considering the motor task and the stimuli that had been presented during the functional tasks in the scanner. Therefore, the foot tapping activations exhibited by ataxic stroke patients with cerebellar damage follow a similar pattern to those seen in other patient populations, indicating their suitability for our patient population. At T2, additional activations of the cerebellum, as well as of the parietal and prefrontal cortex, were revealed, demonstrating the usability of this motor paradigm to monitor changes in lower extremity coordinative function over a three-month period.

**Figure 6:**
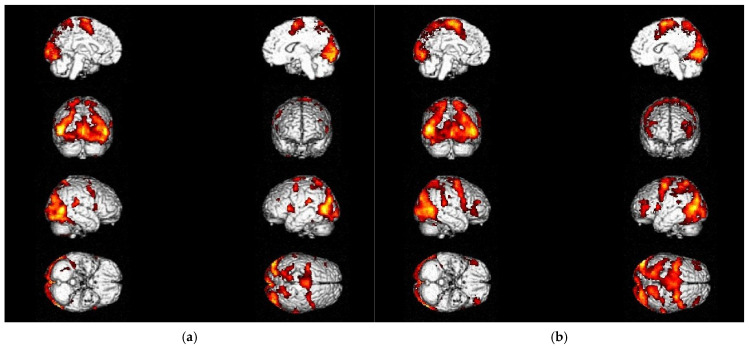
Activation patterns during MI of the balancing task in stroke patients with ataxia: (**a**) at T0; (**b**) at T2.

The MI of the balancing task (P_corr_ < FWE 0.001) at T0 (Table 2) and T2 (Table 3) revealed activation patterns in the PMC, SMA, and cerebellum. Sensory–motor integration areas, interpreting tactile sensory data and the perception of space and limb location (SMG and insula cortex), as well as the dorsolateral prefrontal cortex (dlPFC), were operating during this activity-oriented task as well. Visual and language-relevant areas (visual cortex, FG, Broca Area, and Wernicke area) showed a similar pattern to that observed during the foot tapping task (Figure 6).

The usability to monitor changes over time was also demonstrated for this paradigm, as could be seen by the variability within the results of the measurement timepoints. Significant changes in active brain regions during recovery and rehabilitation are further described in Section 3.2.

### 3.2. Changes in Activation Patterns During Recovery and Rehabilitation

**Figure 7:**
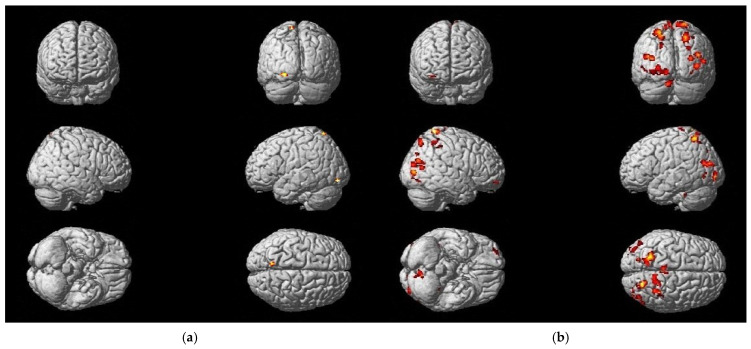
Changes in fMRI activation over the course of recovery and training: (**a**) during foot tapping task—left; (**b**) during foot tapping task—right.

**Figure 8:**
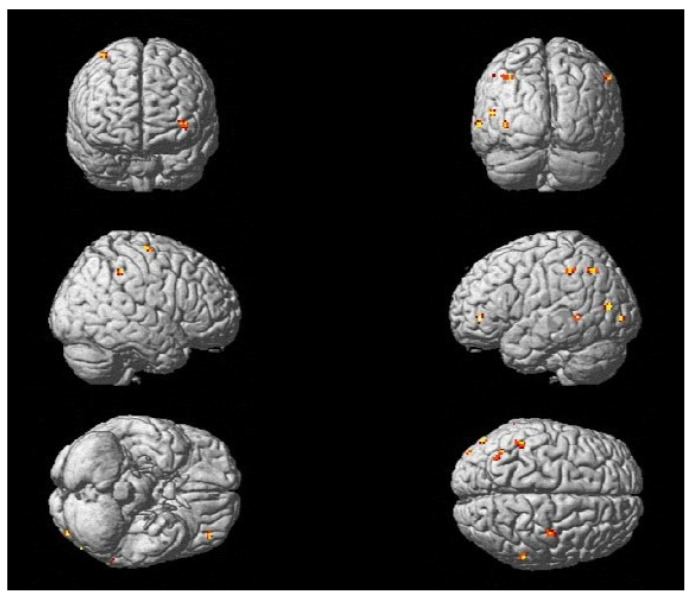
Changes in fMRI activation over the course of recovery and training: during MI of the balancing task (T2 vs. T0).

Analyzing the differences in fMRI activation patterns after 3 months of practice (T2) that had not been present prior to the interventions (T0), Brodmann Area 7 of the parietal cortex was revealed to be the most prominent brain region among all patients in both motor paradigms (Figure 7; Table 4 and Table 5) and in the MI of the balancing task (Figure 8).

At T0, compared to T2, no significant activations above the threshold could be identified for either the foot tapping or the MI of the balancing task. These results suggest an overall increase in brain activity during motor activity and imagination throughout recovery and rehabilitation in our patient population.

When examining the foot tapping task (P_corr_ < FWE 0.05) in more detail, increased activity in the primary motor cortex (M1) and the cerebellum, as well as in the PFC, could be identified after 3 months of practice (T2), suggesting the functional neuronal recovery of the damaged region and its circuits over time (Table 5). These results were consistent with the clinical outcome measures SARA and BBS, which demonstrated significant improvements in ataxic symptoms and balance during the observed rehabilitation period.

For the MI of the balancing task (P_corr_ < FWE 0.05), active regions at T2 (Table 6), besides the parietal cortex, which was prominent among all paradigms of this trial, were motor association areas (SMA and PMC), sensory–motor integration areas (SMG and the middle temporal gyrus, MTG), and the PFC, suggesting the need for increased cognitive resources for this activity-oriented task.

### 3.3. Differences in Activation Due to Treatment Methods

**Figure 9:**
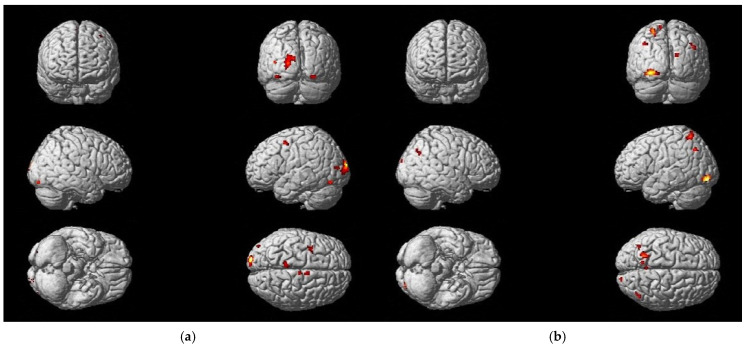
Changes in activation of different brain regions due to treatment method (for foot tapping left): (**a**) the IG showed more active regions at baseline examination (T0 vs. T2); (**b**) the CG demonstrated more active regions after 3 months of practice (T2 vs. T0).

Analyzing the differences in fMRI activation patterns for the foot tapping task (P_corr_ < FWE 0.05) between both treatment groups (IG = Intervention Group; CG = Control Group) that were solely present at T0 (T0 vs. T2) and T2 (T2 vs. T0), the results showed a difference in the course of neuronal plasticity between the two groups (Figure 9), supporting the idea that the effect on neuronal reorganization differs depending on the treatment approach.

In the context of conducting coordination exercises, the IG underwent motor learning in a neuroplastic manner. This was evidenced by a decrease in the activation of associated motor areas (the SMA and PMC) during the training period (Table 7). A greater degree of activation in association and integration areas was evident in the CG after three months of training when compared to the baseline fMRI (T0). Activation was observed in the BA 7 of the parietal cortex and the posterior cingulate cortex (PCC) during the foot tapping task with the left foot, indicating the necessity for compensation through higher sensory integration and additional cognitive resources (Table 8). Analogous findings were observed for the right foot tapping task in the CG (Table S6).

Analyzing the differences in fMRI activation patterns for the MI of the balancing task (P_corr_ < FWE 0.05) between both treatment groups (IG and CG) that were solely present at T0 (T0 vs. T2) and T2 (T2 vs. T0), comparable findings to the foot tapping task could be observed. The IG exhibited wide-spread brain activity at baseline (T0), which became more consolidated at follow-up (T2), whereas the CG showed an increased activation of associated regions at T2.

Additionally, a shift of activation to the lesioned side of the cerebellum could be demonstrated in the IG during the course of training (Tables S7 and S8), suggesting the recovery of damaged pathways. The activation of the cerebellum was observed in the CG as well, albeit still accompanied by prefrontal (ant PFC, BA10) and parietal (BA7) cortex activation after 3 months of training (Table 9).

## 4. Discussion

The aim of this trial was to provide insights into treatment methods for balance and their impact on brain plasticity in the rarely investigated population of acute stroke patients with ataxic symptoms by (1) verifying existing fMRI paradigms (foot tapping and the MI of a balancing task) for their usability in this specific population, (2) following up on neuroplastic changes occurring due to intensive practice over a three-month period, and, finally, (3) by investigating the effects of different approaches in physiotherapy practice on neuronal plasticity.

**Utility of chosen fMRI paradigms in acute stroke patients with ataxic symptoms:** When analyzing movement artifacts, no relation between the severeness of ataxic symptoms and the intensity of the rotational or translational shifting of the head during the scan could be shown. Nevertheless, two patients had to be excluded for the further analysis of the foot tapping task. The MI of the balancing task showed generally fewer movement artifacts. However, one patient had to be excluded for the further analysis of the MI task as well because of inconsistent MI skills, representing in a non-significant single-subject MRI analysis. Nevertheless, both paradigms, foot tapping and the MI of the balancing task, seemed to be equally suitable for this population while displaying active regions of interest (the M1, PMC, SMA, cerebellum, and PFC) in line with our working hypothesis.

**Changes in activation patterns during recovery and rehabilitation:** A very prominent finding in this trial was the consistent activation of Brodmann Area 7 (BA7) of the parietal cortex across all paradigms, indicating an involvement of this area in coordinative functions. BA7 serves as an integration network for various visuomotor programs responsible for reaching, grasping, and bimanual coordination while, in turn, influencing motor areas. This integration may be achieved because networks that are involved in multiple motor programs are merging in this area. This integration network is lateralized to the left hemisphere for unimanual right-hand grasping whereas the right hemisphere specializes in bimanual grasping [36]. The findings of the present trial suggest the contribution of BA7 to sensory–motor integration for lower extremity functions as well, which should be investigated further in trials focusing on coordinative functions. In contrast to the findings of trials investigating upper extremity function, no distinct lateralization for unilateral (foot tapping) or bilateral (balancing task) movements could be observed for lower extremity function in the present population of ataxic stroke patients.

Analysis of the foot tapping task additionally revealed that intensive training over a period of three months resulted in functionally relevant neuronal restructuring, characterized by increased activity in M1 and the cerebellum. In supratentorial strokes, the restoration of function is frequently accompanied by an increased activation of M1 [37,38]. The augmented activation of the M1 region in the leg area, accompanied by the enhanced coordinative function of the lower limbs and increased balance performance, signifies that M1 activation remains a salient indicator of functional recovery and restoration, including balance skills, in ataxic stroke patients with cerebellar damage. Only a few significant activations could be shown in the foot tapping task of the left foot, not including M1 or the cerebellum. These differences in the activation patterns of the foot tapping tasks may be attributable to the unevenly distributed lesion sides within this relatively small population as sensory–motor pathways are known to be lateralized.

Examining the MI of the balancing task, increased activations in motor association areas (SMA and PMC), sensory–motor integration areas (SMG and PMC) and the PFC were revealed after 3 months of training. This result suggests that although patients are capable of performing complex motor tasks at that stage of recovery, they still require the allocation of additional cognitive resources. These increased fMRI activations (SMA and PMC) in ataxic stroke patients with cerebellar damage are different to what is observed in healthy older adults, where balance training leads to a decrease in the activity of the SMA and PMC during the motor simulation of a challenging balance task [35]. This difference could have stemmed from a different baseline level of balance performance as most of the ataxic stroke patients were not able to physically execute tandem walking at the time of baseline examination (T0) while still exhibiting minimal balance impairment after training (T2). Yet, analogous observations have been made when comparing PFC activation during the MI of a balancing task in younger and older adults. In this case, older adults, who have experienced a decline in balance performance, have tended to have augmented PFC activation [39]. Nevertheless, studies have revealed that individuals affected by stroke show decreased function in the right SMG, which is associated with decreased proprioception [40]. Therefore, it can be hypothesized that, in the present study population, the augmented activation of the right SMG was the neuronal correlate of enhanced proprioception resulting from training.

**Differences in activation due to treatment methods:** Analysis of the foot tapping task in the IG, conducting coordination exercises, revealed less activation of associated areas (SMA and PMC) due to training, corresponding to what has been described as motor learning in a neuroplastic manner [41]. In contradistinction, the investigation of the foot tapping task in the CG following a three-month training period revealed heightened activity in associated areas in comparison to baseline fMRI scans (T0). This finding signifies the requirement for compensatory mechanisms involving increased sensory integration (parietal cortex and PCC). The PPC, specifically BA 31, receives spatial and action-related information and influences motor control by relaying information to premotor and supplementary motor areas. Previous trials have shown that BA 31 may be involved in reorganizing brain areas after stroke by facilitating movement through this integration of information [42,43]. Discrepancies in activation patterns between groups may also be attributed to differences in the lesion size and severity of symptoms whereas the IG compromised more patients with moderate symptoms and the CG included more severely affected patients as the course of neuronal recovery is known to differ based on these factors [14].

The analysis of the fMRI results from the MI of the balancing task revealed a propensity for a shift to the lesioned side of the cerebellum in both patient groups. This finding indicates a recovery of motor function, analogous to that observed in patients with supratentorial lesions [44]. Notwithstanding, the CG additionally showed increased activation in associated brain areas (PFC and BA7) after 3 months of training. These results lend support to the outcomes observed in the foot tapping task, revealing slower learning in the CG, as the augmented activation of the motor association areas is known to occur during earlier stages of motor learning [45]. Enhanced frontal control is necessary while movements are still being learned and are not yet automatic, i.e., when patients can only perform tasks with difficulty or with the help of compensation. Similar results have been observed before, where sustained prefrontal activation has been found to be a relevant compensatory mechanism for ataxic gait following an infratentorial stroke [46].

Hence, our findings are in line with the current knowledge that neuronal restructuring and motor learning are still possible in patients with cerebellar damage [17,18,47], albeit with the need for compensation for deficits in trial-and-error learning through specific training strategies. The results of this trial verify the hypothesis that coordination exercises seem to constitute a valid approach to compensate for deficits in this regard. This may be attributed to the high repetition of movement and the fact that patients in the IG demonstrated smaller errors during movement compared to the CG, due to the exercise type (simple vs. complex tasks) as cerebellar patients exhibited an improved ability to learn from errors if they experienced them gradually [20].

**Limitations:** Nevertheless, this study had several limitations to be considered. First, the number of subjects was small, and our results must be confirmed in further studies. Nevertheless, neuronal restructuring in line with the current literature could be shown. Second, the study included patients with distinct sizes and locations of stroke, even though inclusion criteria tried to keep the population homogenous. This represented a limitation as also within the cerebellum, different regions are important for different tasks, i.e., walking or tandem walking [48]. Third, due to the integration of the MRI examination into a larger, already ongoing RCT, randomization into groups lead to an unevenly distributed number of patients in the MRI examination.

## 5. Conclusions

Investigated fMRI paradigms (foot tapping and the MI of a balancing task) were suitable for ataxic stroke patients to monitor changes in neuroplasticity while revealing increased activity in the primary motor cortex (M1) and the cerebellum after intensive training over a three-month period. Coordination exercises, as a specific treatment for ataxic symptoms, have been shown to result in the increased consolidation of brain activity, when compared to the CG, which exposed the augmented activation of association areas, indicating the need for compensatory mechanisms for neuro-motor functions. Activation within Brodmann Area 7 was prominent among all paradigms, implying a special importance of this region for coordinative functions. Due to the limited number of participants and the heterogeneity of the study population, the results must be interpreted with caution. However, they do appear to reflect patterns that are familiar in the neuronal recovery and rehabilitation of stroke patients with supratentorial lesions. Nevertheless, further studies are needed to confirm our results and to transfer them to the overall population of acute stroke patients with ataxia due to cerebellar lesion.

## Figures and Tables

**Table 1:** Clinical characteristics of stroke patients.

	Group	Age	Gender	Location	Lesion Side	BBS T0 *	BBS T2 **	SARA T0 *	SARA T2 **
01	CG	69 a	F	PICA	right	47	56	6	0.5
02	CG	69 a	M	PICA	left	21	55	13.5	2.5
03	CG	58 a	M	SCA and ACP	left	26	54	12.5	6
04	CG	56 a	M	PICA (incl. vermis) and medulla oblongata	left	6	51	20.5	4.5
05	CG	74 a	M	PICA	right	43	53	9.5	6
06	CG	53 a	F	SCA (and pons; asymptomatic)	left	21	48	12.5	9
07	IG	65 a	M	SCA and ponto-mesencephal	left	43	56	7.5	1.5
08	IG	83 a	M	SCA and pons	left	40	54	10	2
09	IG	31 a	F	PICA and SCA and dorsolateral medulla oblongata	left > right	43	56	9.5	2

Abbreviations: IG, Intervention Group; CG, Control Group; a, years old; F, female; M, male; PICA, posterior inferior cerebellar artery; SCA, superior cerebellar artery; ACP, posterior cerebral artery; BBS, Berg Balance Scale; SARA, Scale for the Assessment and Rating of Ataxia. * T0 = Baseline assessment. ** T2 = Assessment after 3 months of practice.

**Table 2:** Activated brain regions during MI of the balancing task at T0 (all participants).

Functional Region	Brodmann Area No.	Cluster		Peak	Coordinates *		
		*p* (FWE-Corr)	No. Voxels	T	X (mm)	Y (mm)	Z (mm)
PMC and SMA left	6	0.000	2449	12.71	−2	−6	62
PMC and SMA left	6	0.000	672	10.34	−30	−8	50
dlPFC left	9	0.000	40	6.58	−44	36	28
V3 left	19	0.000	15651	17.84	−50	−72	2
STG right	22	0.000	480	11.38	66	−30	18
FG left	37	0.000	26	6.70	−42	−40	−20
SMG left	40	0.000	347	10.32	−50	−34	24
Broca left	44	0.000	415	9.25	−50	8	8
Broca right	44	0.000	152	7.19	54	14	14
Broca left	44	0.000	34	6.78	−36	14	10
Cerebellum right	-	0.000	59	6.81	32	−48	−54

Abbreviations: PMC, premotor cortex; SMA, supplementary motor area; dlPFC, dorsolateral prefrontal cortex; V3, tertiary visual cortex; STG, superior temporal gyrus (Wernicke area); FG, fusiform gyrus; SMG, supramarginal gyrus. * Coordinates depict voxel with the highest t-value. FWE: *p* < 0.001.

**Table 3:** Activated brain regions during MI of the balancing task at T2 (all participants).

Functional Region	Brodmann Area No.	Cluster		Peak	Coordinates *		
		*p* (FWE-Corr)	No. Voxels	T	X (mm)	Y (mm)	Z (mm)
S1 right	1	0.000	38	6.56	48	−20	34
Insula right	13	0.000	83	6.87	34	18	8
V3 left	19	0.000	31,363	17.35	−50	−74	8
SMG right	40	0.000	549	10.67	64	−30	20
SMG left	40	0.000	63	7.23	−48	−38	22
Broca left	44	0.000	449	9.31	−50	10	10
dlPFC left	46	0.000	1088	7.51	−44	44	4
Cerebellum right	-	0.000	45	6.82	40	−54	−30

Abbreviations: S1, primary somatosensory cortex; V3, tertiary visual cortex; SMG, supramarginal gyrus; dlPFC, dorsolateral prefrontal cortex. * Coordinates depict voxel with the highest t-value. FWE: *p* < 0.001.

**Table 4:** Changes in activation over time solely present at follow-up: foot tapping—left (T2 vs. T0).

Functional Region	Brodmann Area No.	Cluster		Peak	Coordinates *		
		*p* (FWE-Corr)	No. Voxels	T	X (mm)	Y (mm)	Z (mm)
Parietal cortex left	7	0.001	26	5.68	−12	−62	70
V2 left	18	0.000	63	6.30	−22	−82	−10

Abbreviations: V2, secondary visual cortex. * Coordinates depict voxel with the highest t-value. FWE: *p* < 0.05.

**Table 5:** Changes in activation over time solely present at follow-up: foot tapping—right (T2 vs. T0).

Functional Region	Brodmann Area No.	Cluster		Peak	Coordinates *		
		*p* (FWE-Corr)	No. Voxels	T	X (mm)	Y (mm)	Z (mm)
M1 left	4	0.000	134	5.04	−10	−28	80
Parietal cortex left	7	0.000	320	7.35	−20	−58	54
Parietal cortex right	7	0.000	304	6.90	22	−68	54
Parietal cortex right	7	0.000	194	6.83	20	−44	74
Parietal cortex left	7	0.000	134	6.66	−2	−36	74
Parietal cortex right	7	0.000	138	6.13	34	−48	56
Parietal cortex left	7	0.000	40	5.87	−12	−60	70
Parietal cortex right	7	0.000	33	5.60	28	−78	36
Parietal cortex left	7	0.000	44	5.29	−20	−76	40
PFC right	10	0.001	27	5.48	32	58	−14
V2 right	18	0.000	95	7.14	40	−82	4
V2 left	18	0.000	328	6.32	−12	−86	−4
V3 right	19	0.000	101	6.52	42	−76	24
V3 right	19	0.000	233	6.34	16	−76	2
V3 right	19	0.000	84	6.22	30	−82	16
V3 left	19	0.000	181	6.21	−38	−82	20
V3 right	19	0.000	56	6.02	52	−70	12
V3 left	19	0.001	23	5.33	−46	−72	−4
V3 right	19	0.000	33	5.24	34	−74	−10
FG right	37	0.002	16	5.26	42	−40	−20
Cerebellum left	-	0.000	161	6.15	−8	−72	−24
Cerebellum left	-	0.002	16	5.37	−32	−40	−34

Abbreviations: M1, primary motor cortex; PFC, prefrontal cortex; V2, secondary visual cortex; V3, tertiary visual cortex; FG, fusiform gyrus. * Coordinates depict voxel with the highest t-value. FWE: *p* < 0.05.

**Table 6:** Changes in activation over time solely present at follow-up: MI of the balancing task (T2 vs. T0).

Functional Region	Brodmann Area No.	Cluster		Peak	Coordinates *		
		*p* (FWE-Corr)	No. Voxels	T	X (mm)	Y (mm)	Z (mm)
PMC and SMA right	6	0.001	19	5.58	38	−14	66
Parietal cortex left	7	0.000	26	5.38	−34	−64	46
V2 left	18	0.001	18	5.65	−38	−90	0
V3 left	19	0.000	24	6.03	−50	−78	12
MTG left	21	0.001	17	5.74	−62	−48	2
SMG right	40	0.001	21	5.49	58	−40	44
SMG left	40	0.000	25	5.42	−46	−40	44
dlPFC left	46	0.000	25	5.35	−40	44	0

Abbreviations: PMC, premotor cortex; SMA, supplementary motor area; V2, secondary visual cortex; V3, tertiary visual cortex; MTG, middle temporal gyrus; SMG, supramarginal gyrus; dlPFC, dorsolateral prefrontal cortex. * Coordinates depict voxel with the highest t-value. FWE: *p* < 0.05.

**Table 7:** Changes in activation in in the IG solely present at baseline: foot tapping—left (T0 vs. T2).

Functional Region	Brodmann Area No.	Cluster		Peak	Coordinates *		
		*p* (FWE-Corr)	No. Voxels	T	X (mm)	Y (mm)	Z (mm)
PMC and SMA right	6	0.000	32	5.84	6	−2	64
PMC and SMA right	6	0.001	27	5.71	6	−16	62
PMC and SMA left	6	0.000	38	5.22	−38	6	54
V2 left	18	0.000	219	6.45	−16	−100	18
V2 right	18	0.000	36	6.41	24	−88	−14
V2 left	18	0.001	24	6.02	−8	−84	18
V3 left	19	0.000	48	5.81	−30	−76	−14
V3 left	19	0.001	23	5.75	−40	−86	10

Abbreviations: PMC, premotor cortex; SMA, supplementary motor area; V2, secondary visual cortex; V3, tertiary visual cortex. * Coordinates depict voxel with the highest t-value. FWE: *p* < 0.05.

**Table 8:** Changes in activation in the CG solely present at follow-up: foot tapping—left (T2 vs. T0).

Functional Region	Brodmann Area No.	Cluster		Peak	Coordinates *		
		*p* (FWE-Corr)	No. Voxels	T	X (mm)	Y (mm)	Z (mm)
Parietal cortex left	7	0.000	110	6.22	−24	−58	64
Parietal cortex left	7	0.001	26	5.62	−12	−60	70
Parietal cortex left	7	0.002	15	5.52	−2	−54	52
V2 left	18	0.000	316	7.83	−30	−86	−8
V2 right	18	0.001	24	5.66	16	−98	22
PCC right	31	0.000	110	5.78	12	−58	28
PCC left	31	0.000	33	5.55	−8	−60	20
PCC left	31	0.002	15	5.39	−12	−50	40
AG right	39	0.000	34	5.54	46	−66	36
AG left	39	0.000	31	5.47	−38	−66	42

Abbreviations: V2, secondary visual cortex; PCC, posterior cingulate cortex; AG, angular gyrus. * Coordinates depict voxel with the highest t-value. FWE: *p* < 0.05.

**Table 9:** Changes in activation in the CG solely present at follow-up: MI balancing task (T2 vs. T0).

Functional Region	Brodmann Area No.	Cluster		Peak	Coordinates *		
		*p* (FWE-Corr)	No. Voxels	T	X (mm)	Y (mm)	Z (mm)
Parietal cortex left	7	0.000	467	6.43	−34	−64	46
Parietal cortex right	7	0.000	64	5.58	20	−62	62
Parietal cortex left	7	0.002	16	5.50	−8	−66	52
antPFC left	10	0.001	19	5.46	−26	44	10
V1 right	17	0.001	26	5.51	14	−76	6
V2 left	18	0.000	296	7.91	−36	−92	−6
V2 right	18	0.000	47	6.08	18	−96	20
V3 left	19	0.000	67	6.85	−50	−78	12
V3 right	19	0.000	156	6.36	48	−72	8
FG left	37	0.000	50	6.58	−64	−50	0
FG left	37	0.001	21	5.29	−56	−58	−8
FG left	37	0.000	28	5.09	−44	−64	−20
AG right	39	0.000	31	5.44	30	−76	24
Cerebellum left	-	0.000	46	6.00	−28	−78	−20

Abbreviations: ant PFC, anterior prefrontal cortex; PMC, premotor cortex; SMA, supplementary motor area; V1, primary visual cortex; V2, secondary visual cortex; V3, tertiary visual cortex; FG, fusiform gyrus; AG, angular gyrus. * Coordinates depict voxel with the highest t-value. FWE: *p* < 0.05.

## Data Availability

The data presented in this study is available on request from the corresponding author. The data isnot publicly available due to privacy concerns.

## Supplementary Materials

The following supporting information (Eligibility criteria and activated brain regions) can be downloaded at: https://www.mdpi.com/article/10.3390/neurolint17060084/s1, Table S1: Eligibility criteria of the main trial GLAAS II; Table S2: Activated brain regions during foot tapping—left at T0; Table S3: Activated brain regions during foot tapping—right at T0; Table S4: Activated brain regions during foot tapping—left at T2; Table S5: Activated brain regions during foot tapping—right at T2; Table S6: Changes in activation in Group B (CG): foot tapping—right (T2 vs. T0); Table S7: Changes in activation in Group A (IG): MI of the balancing task (T0 vs. T2); Table S8: Changes in activation in Group A (IG): MI of the balancing task (T2 vs. T0).
