# Supplementary material for: Treatment-Associated Neuroplastic Changes in People with Stroke-Associated Ataxia—An fMRI Study
Source: Neurol Int. 2025 May 29;17(6):84. doi: 10.3390/neurolint17060084 (PMC12196493; doi:10.3390/neurolint17060084)
Supplement: Supplementary file 1 [file neurolint-17-00084-s001.zip › neurolint-3649945-supplementary.pdf]

neurology international

MDPI

# Supplementary Material

**Table S1.** Eligibility criteria of the main trial GLAAS II.

| Inclusion Criteria | Exclusion Criteria |
| --- | --- |
| ≥ 18 years old | modified Rankin Scale (mRS) score ≥ 5 |
| Acute ischemic or hemorrhagic stroke (identified by MRI/CT) | previous stroke leading to persistent debilitating neurological deficits (mRS ≥ 3) |
| Gait ataxia | a comorbidity that limits active study participation (e.g., life expectancy of < 3 months, alcohol or drug abuse) |
| ≥ 1 point on the items gait, stance, trunk or heel-shin-slide of the Scale for the Assessment and Rating of Ataxia (SARA) | Physical or mental conditions that would not allow safe participation in the study or would influence the assessment of outcomes (e.g., dementia, cardiac insufficiency, severe aphasia, etc.), |
| ≤ 47 points on the Berg Balance Scale (BBS) | pregnancy |

**Table S2.** Activated brain regions during foot tapping—left at T0.

| Functional region | Brodmann Area no. | cluster p (FWE-corr) | cluster no. voxels | peak T | Coordinates* x {mm} | Coordinates* y {mm} | Coordinates* z {mm} |
| --- | --- | --- | --- | --- | --- | --- | --- |
| M1 right | 4 | 0.000 | 6597 | 20.76 | 8 | -36 | 74 |
| PMC & SMA right | 6 | 0.000 | 145 | 8.46 | 38 | -6 | 58 |
| PMC & SMA right | 6 | 0.000 | 37 | 6.72 | 56 | 6 | 38 |
| PMC & SMA left | 6 | 0.000 | 85 | 7.61 | -48 | 4 | 4 |
| V2 left | 18 | 0.000 | 5090 | 12.72 | -12 | -92 | 24 |
| V3 left | 19 | 0.000 | 30 | 6.23 | -48 | -72 | 2 |
| FG right | 37 | 0.000 | 47 | 6.66 | 48 | -68 | 2 |
| SMG right | 40 | 0.000 | 609 | 9.22 | 50 | -24 | 24 |
| SMG left | 40 | 0.000 | 144 | 7.39 | -50 | -30 | 22 |
| SMG left | 40 | 0.000 | 37 | 6.56 | -48 | -38 | 32 |
| Broca right | 44 | 0.000 | 111 | 7.67 | 46 | 6 | 8 |

Abbreviations: M1, primary motor cortex; PMC, premotor cortex; SMA, supplementary motor area; V2, secondary visual cortex; V3, tertiary visual cortex; FG, fusiform gyrus; SMG, supramarginal gyrus.

* Coordinates depict voxel with the highest t-value. FWE: p<0.001.

**Table S3.** Activated brain regions during foot tapping—right at T0.

| Functional region | Brodmann Area no. | cluster p (FWE-corr) | cluster no. voxels | peak T | Coordinates* x {mm} | y {mm} | z {mm} |
|---|---|---|---|---|---|---|---|
| S1 left (incl. M1) | 1 | 0.000 | 2589 | 22.44 | -6 | -32 | 68 |
| M1 left | 4 | 0.000 | 33 | 7.38 | -56 | -4 | 40 |
| PMC & SMA left | 6 | 0.000 | 65 | 7.89 | -52 | 2 | 6 |
| PMC & SMA right | 6 | 0.000 | 39 | 6.91 | 58 | 4 | 38 |
| V2 left | 18 | 0.000 | 182 | 9.57 | -18 | -72 | -14 |
| V3 right | 19 | 0.000 | 1682 | 11.19 | 8 | -86 | 34 |
| SMG left | 40 | 0.000 | 366 | 8.37 | -52 | -30 | 24 |
| SMG right | 40 | 0.000 | 128 | 7.16 | 50 | -26 | 22 |

Abbreviations: S1, primary somatosensory cortex; M1, primary motor cortex; PMC, premotor cortex; SMA, supplementary motor area; V2, secondary visual cortex; V3, tertiary visual cortex; SMG, supramarginal gyrus.

* Coordinates depict voxel with the highest t-value. FWE: p<0.001.

**Table S4.** Activated brain regions during foot tapping—left at T2.

| Functional region | Brodmann Area no. | cluster p (FWE-corr) | cluster no. voxels | peak T | Coordinates* x {mm} | y {mm} | z {mm} |
|---|---|---|---|---|---|---|---|
| M1 right | 4 | 0.000 | 6856 | 28.43 | 6 | -34 | 72 |
| PMC & SMA left | 6 | 0.000 | 292 | 9.24 | -48 | 2 | 8 |
| PMC & SMA right | 6 | 0.000 | 133 | 9.18 | 54 | 2 | 38 |
| PMC & SMA left | 6 | 0.000 | 198 | 8.80 | -38 | -10 | 58 |
| PMC & SMA left | 6 | 0.000 | 101 | 7.92 | -50 | -2 | 40 |
| PMC & SMA right | 6 | 0.000 | 63 | 6.59 | 36 | -6 | 58 |
| Parietal cortex left | 7 | 0.000 | 53 | 6.60 | -34 | -46 | 52 |
| V2 left | 18 | 0.000 | 8738 | 14.90 | -8 | -82 | -2 |
| FG right | 37 | 0.000 | 309 | 9.31 | 56 | -62 | -4 |
| SMG left | 40 | 0.000 | 1373 | 12.17 | -54 | -22 | 20 |
| SMG right | 40 | 0.000 | 1495 | 10.31 | 50 | -30 | 32 |
| Broca right | 44 | 0.000 | 295 | 7.70 | 52 | 14 | -4 |
| Cerebellum | - | 0.000 | 67 | 7.38 | 0 | -70 | -34 |
| Cerebellum right | - | 0.000 | 58 | 7.10 | 30 | -60 | -52 |

Abbreviations: M1, primary motor cortex; PMC, premotor cortex; SMA, supplementary motor area; V2, secondary visual cortex; FG, fusiform gyrus; SMG, supramarginal gyrus.

* Coordinates depict voxel with the highest t-value. FWE: p<0.001.

**Table S5.** Activated brain regions during foot tapping—right at T2.

| **Functional region** | **Brodmann** | **cluster** | | **peak** | **Coordinates*** | | |
|---|---|---|---|---|---|---|---|
| | **Area no.** | p (FWE-corr) | no. voxels | T | x {mm} | y {mm} | z {mm} |
| S1 left (incl. M1) | 1 | 0.000 | 22239 | 22.42 | -6 | -30 | 68 |
| PMC & SMA right | 6 | 0.000 | 48 | 6.87 | 54 | 2 | 38 |
| PMC & SMA right | 6 | 0.000 | 59 | 6.31 | 34 | -6 | 54 |
| Parietal cortex right | 7 | 0.000 | 72 | 6.77 | 20 | -70 | 50 |
| aPFC left | 10 | 0.000 | 49 | 6.75 | -38 | 52 | 2 |
| Broca right | 44 | 0.000 | 103 | 6.78 | 54 | 10 | 14 |
| dlPFC right | 46 | 0.000 | 55 | 6.86 | 46 | 42 | 2 |
| Cerebellum right | - | 0.000 | 56 | 7.46 | 24 | -44 | -48 |

Abbreviations: S1, primary somatosensory cortex; PMC, premotor cortex; SMA, supplementary motor area; V2, secondary visual cortex; aPFC, anterior prefrontal cortex; dlPFC, dorsolateral prefrontal cortex.

* Coordinates depict voxel with the highest t-value. FWE: p<0.001.

**Table S6.** Changes in activation in Group B (CG): foot tapping—right (T2 vs. T0).

| **Functional region** | **Brodmann** | **cluster** | | **peak** | **Coordinates*** | | |
|---|---|---|---|---|---|---|---|
| | **Area no.** | p (FWE-corr) | no. voxels | T | x {mm} | y {mm} | z {mm} |
| Parietal cortex left | 7 | 0.000 | 441 | 7.12 | -20 | -58 | 52 |
| Parietal cortex right | 7 | 0.000 | 87 | 6.40 | 20 | -66 | 54 |
| Parietal cortex right | 7 | 0.000 | 67 | 5.60 | 32 | -42 | 52 |
| Parietal cortex right | 7 | 0.001 | 30 | 5.56 | 26 | -78 | 36 |
| Parietal cortex left | 7 | 0.001 | 32 | 5.33 | -14 | -64 | 68 |
| V2 left | 18 | 0.000 | 306 | 6.28 | -34 | -90 | -6 |
| V2 left | 18 | 0.001 | 34 | 5.41 | -10 | -72 | 0 |
| V3 right | 19 | 0.000 | 188 | 6.72 | 20 | -66 | -2 |
| V3 right | 19 | 0.000 | 71 | 6.24 | 52 | -68 | 12 |
| V3 right | 19 | 0.000 | 66 | 6.12 | 42 | -74 | 26 |
| V3 right | 19 | 0.001 | 25 | 5.52 | 42 | -84 | 6 |
| Cerebellum left | 39 | 0.000 | 88 | 6.11 | -8 | -74 | -26 |

Abbreviations: V2, secondary visual cortex; V3, tertiary visual cortex.

* Coordinates depict voxel with the highest t-value. FWE: p<0.05.

**Table S7.** Changes in activation in Group A (IG): MI of the balancing task (T0 vs. T2).

| Functional region | Brodmann | cluster | | peak | Coordinates* | | |
|---|---|---|---|---|---|---|---|
| | Area no. | p (FWE-corr) | no. voxels | T | x {mm} | y {mm} | z {mm} |
| V2 left | 18 | 0.000 | 210 | 6.77 | -20 | -88 | -18 |
| V2 left | 18 | 0.000 | 105 | 6.13 | -6 | -96 | 4 |
| V3 right | 19 | 0.000 | 203 | 8.05 | 28 | -88 | -14 |
| V3 left | 19 | 0.000 | 85 | 7.26 | -50 | -74 | -2 |
| V3 right | 19 | 0.000 | 182 | 6.84 | 44 | -66 | -14 |
| V3 right | 19 | 0.000 | 29 | 6.55 | 46 | -62 | 8 |
| V3 left | 19 | 0.000 | 46 | 6.37 | -38 | -80 | -20 |
| FG left | 37 | 0.000 | 30 | 5.64 | -46 | -42 | -20 |
| FG right | 37 | 0.001 | 25 | 5.38 | 58 | -46 | -24 |
| SMG left | 40 | 0.000 | 37 | 6.17 | -56 | -34 | 28 |
| Cerebellum right | - | 0.001 | 24 | 6.13 | 42 | -52 | -56 |

Abbreviations: V2, secondary visual cortex; V3, tertiary visual cortex; FG, fusiform gyrus; SMG, supramarginal gyrus.

* Coordinates depict voxel with the highest t-value. FWE: $p<0.05$.

**Table S8.** Changes in activation in Group A (IG): MI of the balancing task (T2 vs. T0).

| Functional region | Brodmann | cluster | | peak | Coordinates* | | |
|---|---|---|---|---|---|---|---|
| | Area no. | p (FWE-corr) | no. voxels | T | x {mm} | y {mm} | z {mm} |
| PMC & SMA right | 6 | 0,000 | 51 | 6,45 | 28 | -14 | 70 |
| Cerebellum left | - | 0,000 | 34 | 6,01 | -42 | -44 | -42 |

Abbreviations: PMC, premotor cortex; SMA, supplementary motor area.

* Coordinates depict voxel with the highest t-value. FWE: $p<0.05$.
